# Perceptions of parents and religious leaders regarding minimal invasive tissue sampling to identify the cause of death in stillbirths and neonates: results from a qualitative study

**DOI:** 10.1186/s12978-019-0730-9

**Published:** 2019-05-10

**Authors:** Anam Feroz, Mohsina Noor Ibrahim, Elizabeth M. McClure, Anum Shiraz Ali, Shiyam Sunder Tikmani, Sayyeda Reza, Zahid Abbasi, Jamal Raza, Haleema Yasmin, Khadija Bano, Afia Zafar, Sameen Siddiqi, Robert L. Goldenberg, Sarah Saleem

**Affiliations:** 1 Department of Community Health Sciences, The Aga Khan University, Stadium Road, Box 3500, Karachi, PO 74800 Pakistan; 2 National Institute of Child Health, Karachi, Pakistan; 3 Jinnah Post-graduate Medical Center, Department of Obstetrics and Gynecology-Department of Pathology and Laboratory Medicine, Karachi, Pakistan; 4 Department of Pathology & Laboratory Medicine, The Aga Khan University, Stadium Road, Box 3500, Karachi, PO 74800 Pakistan; 5 RTI International, Durham, USA; 6 Department of Obstetrics and Gynecology, Columbia University, New York, USA

**Keywords:** Minimal invasive tissue sampling, Cause of death, Neonates, Stillbirth, Qualitative study, Parents, Perceptions, Religious leaders, Acceptability, Concerns, Counseling

## Abstract

**Background:**

Recently, the minimal invasive tissue sampling (MITS) procedure has been developed to support determination of the cause of death as an alternate to conventional autopsy, especially in countries where complete diagnostic autopsy is not routine. To assess the feasibility of implementation of the MITS procedure for a study to determine cause of death in premature births and stillbirths in south Asia, we explored the views and perceptions of parents and religious leaders on the acceptability of MITS.

**Methods:**

A qualitative study was conducted at the National Institute of Child Health (NICH) hospital of Karachi, Pakistan. Focus group discussions (FGDs) were conducted with parents of newborns who visited well-baby clinics of the NICH hospital for post-natal check-ups. Key-informant interviews (KIIs) were conducted with religious leaders. Data were analyzed using NVivo 10 software.

**Results:**

A total of 13 interviews (FGDs = 8; KIIs = 5) were conducted. Three overarching themes were identified: (I) acceptability of MITS; (II) concerns affecting the implementation of MITS; and (III) religious and cultural perspectives. Participants’ acceptance of MITS was based on personal, religious, cultural and social beliefs. Parents widely recognized the need for this procedure in cases where the couple had experienced multiple stillbirths, neonatal deaths and miscarriages. Counseling of parents was considered vital to address emotional concerns of the parents and the family. Religious leaders indicated acceptability of the MITS procedure from a religious perspective and advised that respect for the deceased and consent of the guardians is mandatory when performing MITS.

**Conclusions:**

This qualitative study provided a unique opportunity to understand the views of parents and religious leaders towards the use of MITS. Generally, MITS appears to be an acceptable method for identifying the cause of death in neonates and stillbirths, provided that the deceased is respected and buried as soon as possible without any delays and parents are counseled appropriately. Findings from this research are essential in approaching families for consent for MITS.

## Plain English summary

Recently, the minimal invasive tissue sampling (MITS) procedure has been developed to help inform the cause of child death. Qualitative studies also suggest that MITS appears to be acceptable and technically feasible because it is non-disfiguring, less difficult, and inexpensive and can be performed quickly, thus fewer delays for the family and potential burial practices. However, such findings may not be relevant for a country like Pakistan, where 96% of the total population is Muslim. Therefore, we undertook a study to explore and understand the acceptability of MITS among parents, and religious leaders in Pakistan. Interviews were conducted with parents of newborns who visited well-baby clinics of the NICH hospital for post-natalcheck-ups and with religious leaders. Parents widely recognized the need for this procedure in cases where the couple had experienced multiple stillbirths, neonatal deaths and miscarriages. Counseling of parents was considered vital to address emotional concerns of the parents and the family. Religious leaders indicated acceptability of the MITS procedure and advised that respect for the deceased and consent of the guardians is mandatory when performing MITS. Findings from this research are essential in approaching families for consent for MITS.

## Background

In low-and-middle-income countries (LMICs), verbal autopsies (VA) are the most common technique for investigating the cause of child death [1]. However, studies indicate that VA methodology is often inaccurate and has poor specificity [2]. As a consequence, there remain high uncertainties in regional and national estimates of causes of child mortality due to poor cause of death (CoD) investigations [3]. The feasibility and acceptability of complete diagnostic autopsy (CDA), the gold standard for CoD determination, faces noticeable challenges and acceptability issues [4]. These include resource constraints, fear of body disfigurement and cultural and religious criticisms [5]. Recently, the minimal invasive tissue sampling (MITS) procedure has been developed to help inform the CoD determination. This procedure involves body inspection and recording of basic anthropometric data, body palpation, body imaging and biopsy to obtain samples of lungs, brain, liver and other organs for histopathologic and microbiologic examination rather than extensive examination of internal organs [4, 6].

Preliminary findings of a multicenter research project to directly compare MITS with full-autopsy found that MITS has a good diagnostic concordance with CDA, and is an acceptable alternative to full autopsy for CoD determination [7]. Qualitative studies also suggest that MITS appears to be acceptable and technically more feasible than the CDA because it is non-disfiguring, less difficult, and inexpensive and can be performed quickly, thus fewer delays for the family and potential burial practices [8–11]. However, such findings may not be relevant for a country like Pakistan, where 96% of the total population is Muslim [12, 13]. In addition, MITS may function differently across countries with diverse health care systems. Therefore, it is important to explore parents’ and religious experts’ perceptions of the MITS procedure and their decision-making processes within the specific context as the procedure involves several technical, religious and cultural challenges.

To address these concerns, we undertook a study to explore and understand the acceptability of MITS among parents, and religious leaders in Pakistan [10]. This study was conducted in preparation for a larger study, the Project to Understand and Research Preterm Pregnancy Outcome in South Asia (PURPOSe). For PURPOSe, MITS will be performed on both stillbirths and preterm neonates to help determine the CoD [14]. Before the implementation of PURPOSe, there was uncertainty about how parents’ would perceive MITS and whether it would be considered acceptable in places where the Muslim religion was predominant.

## Methods

### Study design and setting

We used an exploratory qualitative research design to collect data from the purposively sampled population as described in detail elsewhere [10]. The study was conducted at the National Institute of Child Health (NICH) hospital, because of their well-established pediatric care protocols and willingness to participate in the study. FGDs were conducted at the outpatient department (OPD) and well-baby clinics of the NICH hospital, while KIIs were conducted at the respective locations of key-informants.

### Study participants

Parents of newborns who were visiting the OPD and well-baby clinics of NICH hospital for regular growth monitoring, post-natalcheck-ups and vaccinations were purposively sampled for focus group discussions (FGDs). Considering the cultural and ethical sensitivity, the study excluded parents and/or families that experienced a recent neonatal death/stillbirth or who were in-patients. Religious leaders, including Sunni Ulemas and Shia Muftis were purposively sampled for key-informant interviews (KIIs). In addition, we interviewed health care providers with results presented elsewhere.

### Interview guide

Separate semi-structured interview guides for FGDs and KIIs explored participants’ views and attitudes towards MITS, with focus on the cultural and religious perspectives, perceived benefits, concerns affecting implementation and factors facilitating the implementation. Before beginning the interview, the qualitative researchers first described an overview of autopsy and the MITS procedure (Table 1).

**Table 1:** Overview of open autopsy and MITS procedure

Full autopsy - Body inspection and recording of basic anthropometric data; body weight, height/length, mid-upper arm circumference, head circumference, lower leg length and foot length - Extensive examination of internal organs begins with the creation of a Y or U- shaped incision from both shoulders joining over the sternum and continuing down to the pubic bone	
MITS - Body inspection and recording of basic anthropometric data; body weight, height/length, mid-upper arm circumference, head circumference, lower leg length and foot length - Body palpation by a MITS specialist. - Imaging/photography by a MITS technician - Biopsy needles to obtain samples of lung, brain, liver and other organs for histopathologic and microbiologic examination to help determine COD	

### Data collection

A free flow of information was encouraged, using probes from these discussions to obtain parents’ perception of the MITS procedure. Interviews were conducted face-to-face in Urdu or English according to the participants’ preferences and were audio recorded with consent from study participants. Detailed field notes were also taken during each interview to capture non-verbal language and cues. KIIs lasted between 20 and 40 min; FGDs lasted 30 min to 1 h and consisted of five participants per group.

### Analysis

Study data were analyzed using thematic analysis facilitated by NVivo version 10 (QSR International, Pty Ltd) software. Data were collected and analyzed through an iterative process and data collection was ceased once saturation was achieved. First, the audio recordings were transcribed into English and then the transcriptions were uploaded in the software. Transcripts were read several times to develop an interpretation of the participants’ perception towards MITS. Focus groups and KIIs were coded as two data sets. Two investigators coded a subset of transcripts independently using separate NVivo files which were then combined to match codes, and agreement was sought on a coding framework. The remaining transcripts were then independently coded according to the framework. Codes were formulated inductively from the transcripts. Coding discrepancies were discussed and resolved to reduce researcher’s bias. Codes were then analyzed under major themes. Semi-structured interview guides were used to outline major themes. Final overarching themes were discussed and reviewed by both investigators. To ensure the credibility of the research, study data were triangulated by the data sources (mothers, fathers, religious leaders) and data collection methods (FGDs and KIIs), to compare alternative perspectives and assess any inconsistencies.

### Ethical considerations

Ethical approval for this study was obtained from the NICH hospital [11/2018] and the Aga Khan University Ethical Review Committee (AKU-ERC) – [5358-CHS-ERC- 18]. Written informed consent was provided by all study participants. Informed consent included permission to audio record the interviews and use anonymized quotes.

## Results

In this qualitative study, eight FGDs and five KIIs were conducted with a variety of stakeholders (parents and religious leaders), between July 2018 and August 2018, to ascertain the acceptability of MITS to identify CoD among stillbirths and neonates (Table 2). The demographic information for the FGD participants is illustrated in Table 3. All the study participants (*n* = 45) who were approached by the study team agreed to participate in the study. All the respondents were Muslim.

**Table 2:** Study Participants

Focus Group Discussions with Parents	Total FGDs =8; *n* = 40
Mothers of newborns who were visiting OPD and well-baby clinics of NICH hospital for regular post-natal check-ups	4 groups (5 participants in each group)
Fathers of newborns who were visiting OPD and well-baby clinics of NICH hospital for regular post-natal check-ups	4 groups (5 participants in each group)
Key Informant Interviews with Religious Leaders	Total KIIs = 5; *n* = 5
Sunni Ulemas	3 KIIs
Shia Muftis	2 KIIs

**Table 3:** Demographic information for the FGD study participants

Pseudonym	Age	Gender	Socio-economic status	Religion	Education	Employment
FGDs with mothers of new-borns who were visiting OPD and well-baby clinics of NICH hospital for regular post-natal check-ups						
FGD 01						
FGD01-W01	32	F	Lower	Muslim	Grade 5	Unemployed
FGD01-W02	24	F	Lower	Muslim	None	Unemployed
FGD01-W03	33	F	Lower-middle	Muslim	None	Unemployed
FGD01-W04	27	F	Lower	Muslim	None	Unemployed
FGD01-W05	26	F	Lower	Muslim	Grade 8	Unemployed
FGD 02						
FGD02-W01	27	F	Lower-middle	Muslim	None	Unemployed
FGD02-W02	38	F	Lower	Muslim	None	Unemployed
FGD02-W03	42	F	Lower	Muslim	Grade 6	Unemployed
FGD02-W04	26	F	Lower-middle	Muslim	None	Unemployed
FGD02-W05	36	F	Lower	Muslim	Grade 8	Unemployed
FGD 03						
FGD03-W01	29	F	Lower	Muslim	None	Unemployed
FGD03-W02	32	F	Lower	Muslim	None	Unemployed
FGD03-W03	22	F	Lower-middle	Muslim	Grade 2	Unemployed
FGD03-W04	34	F	Lower	Muslim	None	Unemployed
FGD03-W05	37	F	Lower	Muslim	None	Unemployed
FGD 04						
FGD04-W01	19	F	Lower	Muslim	None	Unemployed
FGD04-W02	38	F	Lower	Muslim	None	Unemployed
FGD04-W03	23	F	Lower-middle	Muslim	Grade 3	Unemployed
FGD04-W04	35	F	Lower-middle	Muslim	Diploma	Employed
FGD04-W05	28	F	Lower	Muslim	None	Unemployed
FGDs with fathers of new-borns who were visiting OPD and well-baby clinics of NICH hospital for regular post-natal check-ups						
FGD 05						
FGD05-M01	39	M	Lower	Muslim	None	Employed
FGD05-M02	45	M	Lower	Muslim	Grade 7	Employed
FGD05-M03	32	M	Lower-middle	Muslim	None	Employed
FGD05-M04	31	M	Lower	Muslim	None	Employed
FGD05-M05	48	M	Lower	Muslim	None	Employed
FGD 06						
FGD06-M01	36	M	Lower	Muslim	None	Employed
FGD06-M02	29	M	Lower	Muslim	Grade 3	Employed
FGD06-M03	34	M	Lower	Muslim	None	Employed
FGD06-M04	47	M	Lower	Muslim	None	Employed
FGD06-M05	33	M	Lower-middle	Muslim	None	Employed
FGD 07						
FGD07-M01	27	M	Lower	Muslim	None	Employed
FGD07-M02	36	M	Lower	Muslim	Grade 1	Employed
FGD07-M03	42	M	Lower	Muslim	None	Employed
FGD07-M04	28	M	Lower	Muslim	None	Employed
FGD07-M05	41	M	Lower	Muslim	None	Employed
FGD 08						
FGD08-M01	26	M	Lower	Muslim	None	Employed
FGD08-M02	39	M	Lower-middle	Muslim	None	Employed
FGD08-M03	47	M	Lower	Muslim	None	Employed
FGD08-M04	32	M	Lower	Muslim	Grade 6	Employed
FGD08-M05	38	M	Lower	Muslim	None	Employed

Based on the data collection and thematic analyses, three overarching themes were identified (Table 4): (I) acceptability of MITS; (II) concerns affecting the implementation of MITS; and (III) religious and cultural perspectives of the KIIs. These themes are presented below with illustrative quotes

**Table 4:** Themes and sub-themes

Theme	Sub-theme
Acceptability of MITS	Likely uptake in recurrent pregnancy loss
	Willing to know the cause of death of a loved one
	Doesn’t bring the dead back to life
	Perceived societal benefit
	Socio-demographic factors influencing the acceptance of procedure
	Fear of unexpected medical findings
	Counseling for MITS
Concerns affecting implementation of MITS procedure	State of mind around death
	Disrespect for the deceased
	Possible delays to funeral
	Consent and decision making
Religious and cultural perspective	Religious permissibility
	Compelling justification for performing the procedure
	Seeking permission of the guardian

### Focus group discussions with parents

#### Acceptability of MITS

##### Likely uptake in recurrent pregnancy loss

Generally, most parents showed a positive attitude towards the MITS procedure. Parents widely recognized that there likely will be uptake for MITS procedure in cases where parents have experienced multiple neonatal deaths, stillbirths or miscarriages.

“I know a lady who has lost her four or five children and she still does not know the cause of death. In such cases, a mother will opt for MITS to know the cause of her child’s death.” (Mother, FGD)

Most parents expressed willingness to consent for MITS as it would inform them about the possible reasons that led to the child’s death. Parents verbalized that they would want to know the CoD so that remedial actions could be taken to prevent deaths in subsequent pregnancies.

“Many parents would give permission for this procedure as it would help them prevent further deaths in subsequent pregnancies.” (Mother, FGD)

##### Willing to know the cause of death of a loved one

Among most mothers, the potential acceptability of the MITS procedure was associated with their willingness to know the CoD of a loved one. One mother mentioned that a few years back she experienced the sudden death of her child and would have requested this procedure to understand what went wrong at the ninth month of pregnancy.

“My first baby was born dead… Four days before the delivery, I got to know that the baby has died… I wanted to know what happened… I would have gone for MITS because my baby was perfectly fine for the whole nine months. I came for the checkups, doctors told me my baby is doing perfectly fine, and they gave me an expected date of delivery. Then something wrong happened suddenly. At least I could have explored what went wrong.” (Mother, FGD)

Fathers discussed that most people in the city are not cognizant about the methods that can help identify the cause of child death. They believed that most parents would agree for the procedure if they would be informed that non-invasive option is available.

“There are many people here in cities that are not aware of what can be done to identify the cause of death. If you will tell them, they will allow you to find out what went wrong.” (Father, FGD)

##### Doesn’t bring the dead back to life

A few mothers verbalized that after death there was no hope and nothing which could help the deceased child. Therefore, most parents would not be willing to undertake any such procedure. Several fathers also mentioned that parents would not provide permission as MITS could not bring back their deceased child, as illustrated in the following quote:

“This would be a very emotional situation for the mother, because she has lost her child keeping him/her in her womb for months. She will never agree as it would not help her bring back the deceased child. If I was at her place I would have never agreed for this.” (Mother, FGD)

##### Perceived societal benefit

Most parents reported that they would be willing to consent for MITS if it could save the life of someone else’s child in the future. For several mothers, the procedure was seen as beneficial for the community and society in general as it has the potential to provide better knowledge about the diseases and treatments.

“If my child can save someone else’s child then yes definitely, this should be done.” (Mother, FGD)

##### Socio-demographic factors influencing the acceptance of the procedure

Most parents verbalized that acceptance highly depends on one’s religious beliefs. Several parents mentioned that they would like to know the religious interpretation of these procedures and expressed a desire to consult religious leaders to know whether such procedures are permissible in Islam.

“This depends on what religious people have to say, because we will have to see if Islam gives permission for this. If Islam permits then people will also support you… if it is in our good faith, then this should be done.” (Father, FGD)

In addition to religious beliefs, parents believed that education and socio-economic status (SES) are also important factors that determine acceptance for MITS procedure. Some mothers indicated that unfortunately most child deaths occur in impoverished families where death is viewed as nature’s decision. A few fathers reported that only educated families and parents would understand the need for such procedures.

“In my opinion, if the family has an educated background, they would show willingness for MITS procedure. If they do not have that capacity, they will consider death as Allah’s will.” (Father, FGD)

##### Fear of unexpected medical findings

A few mothers believed that most women would not consent because of the fear of unexpected medical findings. Mothers verbalized that for most women, it would be really difficult to agree for this procedure because if something unexpected is discovered during the procedure, the husband and in-laws would blame them. Mothers expressed acute anxiety with the thought that their marriage could end or their husbands would opt for a second marriage.

“For a mother, there’s a fear that if she would go for the tests and something unexpected comes out, the husband and in-laws will trouble her.” (Mother in an FGD)

##### Counseling for MITS

Mothers acknowledged that effective counseling is crucial for the acceptability of the MITS procedure. Parents and religious leaders widely recognized the role of trained counselors who are mindful of common cultural and religious concerns when discussing MITS with parents/families. Moreover, parents emphasized that religious leaders should be part of the counseling team as they would help address religious concerns. A few parents suggested that the hospital team should first communicate with the elders of the family as they can better convince the parents of the need to determine the CoD of the deceased child.

“A parent is so emotional at that time …I am saying this because I have been through this traumatic situation. If somebody could convince him for this procedure, that could be his parents (grandparent of the deceased child).” (Father, FGD)

### Concerns affecting implementation of MITS procedure

#### State of mind around death

Parents verbalized that the family’s state of mind around child death is an important concern affecting acceptance and implementation of MITS. Mothers reported that it will be challenging to approach a family for MITS immediately after the death as they will be experiencing acute grief. Most mothers suggested that physicians should be very empathetic and gentle with the parents of the deceased and should be able to respond to their concerns.

“This would be a very emotional situation for the mother as she has lost her child after keeping him in her womb for months. She will never agree. If I was at her place, I would have never agreed for this.” (Mother, FGD)

#### Disrespect for the deceased

Despite the MITS procedure potentially being religiously acceptable, some parents stated that they would decline consent for this procedure since it is considered disrespectful in their culture. Moreover, they believed that death was meant to happen as it was Allah’s will and therefore they would not want to know the CoD.

“See this is very difficult for parents. They would not allow for this procedure even if it is religiously acceptable. Many parents would find it disrespectful.” (Father, FGD)

#### Possible delays to funeral

A few parents believed that the MITS procedure could delay the funeral. Fathers indicated that especially families from distant places are likely to decline the procedure as they would like to go back to their villages soon after the death to carry out religious customs surrounding burial. Mothers mentioned that parents would not be willing for any such procedure and would want to bury their deceased child as soon as possible without any delays. As one mother in FGD explained:

“If Allah has given me the child and then took it back from me, I would want to bury my child as soon as possible*.*” (Mother, FGD)

#### Consent and decision making

Social relationships were another factor that affects the consent and decision-making process for MITS. Most mothers verbalized that in a patriarchal marriage, females have little control or influence over such situations and the husbands are authorized to make final decisions about such procedures. In some cases, grandparents are given authority for such decisions as they are perceived to have a mature perspective. Most mothers mentioned that even if they provided consent for the MITS procedure, the society and family members would humiliate them and impede their participation, as illustrated by the following quote:

“Even if a mother would agree for MITS, elders of the family would say she is doing this kind of thing with her child.” (Mother, FGD)

### Key informant interviews

#### Religious and cultural perspectives

##### Religious permissibility

Most religious leaders expressed concerns related to the extent to which the deceased would be touched and affected. The religious key-informants mentioned that the dead body should be examined very carefully during the procedure so that it has minimal damage. One religious leader suggested that the procedure should be rigorously followed to avoid any further mutilation.

“If you can do the samplings from the body with minimum damage, then there is no restriction from Shariah. However, on the conditions that the respect of the body is considered and not going any further of what you basically require.” (Religious Leader, KII)

##### Compelling justification for performing the procedure

Most of the religious leaders believed that MITS would only be permissible if there is a compelling justification to perform the procedure. All religious leaders noted that without any persuasive reason, mutilating a dead body is forbidden in Islam and that the dead body should be given utmost respect and dignity. Many religious leaders voiced their opinion that the dead body could still feel pain and therefore, respect for the deceased is mandatory.

“According to Shariah of Prophet Mohammed (P.B.U.H), the respect for the body after death is the same as it is during life… Respect for the body after death is slightly greater, and touching a living or dead body without any valid reason is strongly prohibited.” (Religious leader, KII)

##### Seeking permission of the guardian

Religious leaders emphasized that obtaining the permission of the guardian before performing the procedure is mandatory. The respect for the body is mandatory in Islam and the guardian bears the responsibility of ensuring that the deceased is fully respected. The religious leaders recommended that before initiating any such procedure, the first step would be to take consent from the guardian, as illustrated by the following quote:

“The first law is the respect of the body, and the rightful heir bears the responsibility of the respect of body… If we had to do some research for which we require touching or looking at the dead body to find out what went wrong, then the first step is to seek permission of the rightful heir.” (Religious leader, KII)

## Discussion

This study identified the factors influencing the acceptability of MITS among Muslim parents in Pakistan. Generally, MITS appears to be an acceptable method for identifying the CoD in neonates and stillbirths. According to a previous study, the procedure has received resistance from the Muslim communities [13]. However similar findings were reported in a socio-behavioral study conducted in Africa and South Asia to understand the feasibility and hypothetical acceptability of MITS. The research found that majority of the Muslim participants interviewed would be willing to know the CoD (78%) and would accept MITS (70%) [15]. Our study further informs that the factors influencing the acceptability of MITS are complex and multifaceted in the context of Pakistan as most of the population is Muslim. Participants’ insights regarding the perceived acceptability of MITS were based on personal, religious, cultural and social beliefs.

The majority of the parents widely recognized the need for this procedure for couples who have experienced multiple neonatal deaths, stillbirths and miscarriages. They also articulated that the parents who have experienced loss of a baby would grant permission for MITS to establish the cause of child’s death. Interestingly, these viewpoints align with those in the Lewis et al. study where FGD participants indicated acceptance for the less invasive autopsy if the family had experienced multiple fetal losses [16]. Most importantly, the study suggested that the high hypothetical acceptability of MITS was due to parent’s own willingness to know the cause of child death. With reference to the acceptance of MITS, counseling for the parents was considered vital to address emotional concerns. Previous studies have similarly reported that providing complete information and support to the families through counseling is crucial for the acceptability of MITS among parents [4, 9]. Another enabling factor for the uptake of MITS was the procedure’s potential to benefit the society and community at large. Finally, parents’ educational status was reported as a likely contributor to the acceptance for MITS procedure as educated families are believed to be better able to understand the need for such procedures.

A number of concerns affecting the implementation of the MITS procedure were identified in the study. These included parent’s state of mind around the time of the death, possible delays to the funeral, perceived disrespect of the deceased and fear of unexpected medical findings, consistent with the previous research findings [16].

Social relationships can play a major role in the uptake of MITS. Previous studies have also recognized that social relations, power and gender dynamics are crucial to acceptance for MITS [17]. While most participants reported that mothers-in-law and elders are the decision-makers, in some cases, fathers are the primary decision-makers over matters involving the deceased child. It can be argued that women’s lack of decision-making power could affect the uptake of MITS. Therefore, it is necessary to advocate for the rights of mothers and wives to consent for MITS [17]. Similar to our study, a study in Bangladesh suggested that women would be unable to refuse MITS if their partners agreed and therefore, unequal power relationships could coerce participants to agree against their will. This study concludes that investigators should be aware of potential harms and should address them through corrective actions [5].

Religious and cultural perspectives emerged as the main theme, which was perceived both as a facilitator and a barrier towards the acceptability of MITS. Contrary to preconceptions on this subject, Muslim leaders such as Shia Muftis and Sunni Ulemas, from the two major sects of Islam, indicated high acceptability for the MITS procedure. To address the religious concerns, ensuring that there is respect for the deceased, no mutilation to the body and permission of the guardian when there was reasonable justification were the main factors to increase acceptability of MITS. Most parents argued that religious leaders, in particular Muslim leaders, should determine the acceptability of MITS, similar to reports from studies in other Muslim societies [5].

Several parents believed that the body is sacred after death and should not be disrespected by any means. They believed that the deceased should be buried as soon as possible after death, with all burial rituals similar to other studies [9]. This indicates the need for ensuring that the trained counselors and health care providers are mindful of the common religious and sociocultural beliefs when discussing MITS with parents/families.

While the findings of this study are promising for the potential uptake of MITS for CoD determination, they remain theoretical and will be validated with data regarding the acceptance during the implementation study. Yet, there are some study findings which have influenced the manner in which consent is obtained in the main study (PURPOSe). These include first, that the primary physician team should introduce new members of the MITS team to the patient/family; counsellors should seek consent, primarily from parents in-person and in a quiet and private setting; counselors should understand MITS procedure from a religious perspective; and counsellors should describe the MITS procedure like a biopsy technique. The major limitation of this research is that the acceptance of MITS was explored with the parents in a hypothetical situation.

## Conclusion

This qualitative study provided an opportunity to explore the views of parents and religious leaders towards MITS. In general, parents viewed the MITS procedure positively when it is performed with respect of the deceased. Findings from this research will be essential in providing appropriate counseling and consent for MITS. Ultimately to understand the feasibility and acceptability of MITS in Pakistan, close follow-up of study participants is needed to understand the consent and decision-making process, and explore factors that could affect the acceptance for MITS. This exploratory qualitative study could also be replicated in settingswhere preliminary feasibility and acceptability of MITS is not ascertained among parents, families and religious leaders.

## Abbreviations

CDA: Complete Diagnostic Autopsy
CoD: Cause of Death
ERC: Ethical Review Committee
FGDs: Focus Group Discussions
KIIs: Key-informant Interviews
LMIC: Low-and-middle-income-countries
MITS: Minimal Invasive Tissue Sampling
OPD: Outpatient Department
VA: Verbal Autopsies
